# The Consistency of Primary, Secondary and Tertiary Prevention Definitions in the Context of Musculoskeletal Sports Injuries: A Rapid Review and Critical Exploration of Common Terms of Usage

**DOI:** 10.1186/s40798-025-00823-y

**Published:** 2025-03-18

**Authors:** Aske Holm-Jensen, Evgenios Vlachos, Louise Kamuk Storm, Corrie Myburgh

**Affiliations:** 1 Department of Sports Science and Clinical Biomechanics, University of Southern Denmark, Campusvej 55, Odense M, Denmark; 2 University of Southern Denmark, University Library of Southern Denmark, Odense M, Denmark; 3 University of Southern Denmark, The Maersk Mc-Kinney Moller Institute, Odense M, Denmark; 4 The Chiropractic Knowledge Hub, University of Southern Denmark, Odense M, Denmark; 5 Department of Chiropractic, University of Johannesburg, Johannesburg, South Africa

**Keywords:** Sport injury, Athletic injuries, Prevention Nomenclature, Prevention classification, Prevention definition, Prevention aim, Prevention objective

## Abstract

**Background:**

Formal statements articulating the meaning of primary, secondary and tertiary prevention concepts are commonly used in the musculoskeletal sports injuries literature, but appear to be employed inconsistently and incorrectly. Standard definitions, appropriate to athletic health and performance practice, are required to systematically develop the state-of-the-art. To accomplish this, we summarized prevention definitions with the aim of improving conceptual clarity across the musculoskeletal sports injuries literature.

**Main body:**

We used a rapid literature review method, searching Scopus, PubMed/Medline, Cochrane Library reviews/trials, Web of Science, Sports Medicine and Education Index, SPORTDiscus and CINAHL databases for titles/abstracts for available literature, published in English from database-inception to November 2023. Our search terms were: sport/athlete, injury, primary prevention, secondary prevention, and/or tertiary prevention. Definitions were extracted to create categories illustrating overlap and variation. We extracted definitions from 144 included studies (n). Primary prevention appears focused on mitigating injury risk (*n* = 52) and preventing initial injuries (*n* = 42). Secondary prevention appears to address five distinct concepts: preventing recurrences (*n* = 42), preventing sequelae (*n* = 41), preventing index injury worsening (*n* = 27), mitigating injury risk (*n* = 15), and restoring function (*n* = 12). Tertiary prevention appears focused on preventing sequelae (*n* = 17) and restoring function (*n* = 9).

**Conclusions:**

From a definition viewpoint, the aim of primary prevention is narrowly conceptualized and consistent in the musculoskeletal sports injury research literature. However, secondary prevention definitions vary substantially, with at least three distinct conceptual aims observable. Tertiary prevention definitions appear infrequently in the literature and when observed tend to overlap with secondary prevention. Currently, researchers are likely to struggle with the formulation of clearly-defined and transferrable research questions relating to the aims of secondary prevention.

**Supplementary Information:**

The online version contains supplementary material available at 10.1186/s40798-025-00823-y.

## Key Points

Substantial heterogeneity in the choice of definition and reporting of primary, secondary and tertiary prevention was evident in the musculoskeletal sports injuries research literature.

The aim of primary prevention is preventing the initial injuries and mitigating risk factors.

Secondary prevention in the musculoskeletal sports injuries literature is inconsistently defined. Definitions cover preventing sequelae, preventing recurrences, preventing worsening, mitigating risk and restoring function.

We suggest the aim of secondary prevention is defined as preventing sequelae, preventing recurrences, and preventing worsening.

Tertiary prevention appears infrequently in the literature, and tends to overlap with secondary prevention. We suggest that the aim of tertiary prevention is defined as restoring function.

## Background

In preventive health care, interventions are conceptually labelled primary, secondary or tertiary preventive strategies [1]. Initially promulgated in epidemiology, these terms are now widely observable across all facets of the health care landscape as they offer a means for readily distinguishing the perceived focus of any particular intervention [2]. Briefly stated, primary prevention aims to address a health care issue before exposure is detected (preventing a disease from occurring), secondary prevention aims to address the health care issue after exposure is detected, but before clinical sequelae are evident (screening of asymptomatic persons with a view to early detection and treatment of disease), and finally tertiary prevention aims to ameliorate the clinical sequelae that may already have occurred (treatment of patients with a view to palliation, cure, rehabilitation, prevention of relapse, or prevention of complications) [1, 3].

This classification system, despite its popularity, is not without issues and has been criticized for being too non-specific to allow scholars to frame operational definitions consistently [1]. Based on systematic interrogation of definitions, Froom & Benbassat advocated for the use of more specific terms of reference when operationalizing prevention (intervention objective, type and target population), rather than the use of a meta-concept open to individual interpretation [4].

In the context of athletic health and performance management, minimizing risk (rather than detecting exposure) and rehabilitating acute injuries occupy a central position in the pursuit of strategic goals. Thus, as seen in recent models describing the classes of prevention in this context, descriptions of primary, secondary and tertiary prevention reflect this conceptual bias [5]. As a consequence, primary prevention refers to interventions occurring in the absence of a health risk/problem, secondary prevention occurs in the presence of a health risk, but with no apparent problem (diagnosis), and thirdly tertiary prevention occurs when both a health risk and persistent problem have been detected [6].

To our knowledge, a study, systematically investigating the consistency of prevention class definitions in the context of musculoskeletal sports injuries, has not been performed. However, our preliminary reading on the topic is suggestive of a similar tendency observed by Froom et al. [1]. For example, there is disagreement between researchers whether to define primary prevention as index injury avoidance or risk mitigation [7]. Moreover, secondary prevention has been interchangeably defined as the prevention of recurrences [8, 9], the prevention of injury sequelae [10, 11], and the early symptom identification and therapeutic intervention [12, 13]. Consequently, prevention definitions appear to straddle several concepts, which complicates the articulation and development of interventions targeting the three prevention classes (see Table 1 for additional examples of common terms).

**Table 1:** Common terms in the sports injuries literature, classified according to the referenced papers’ definition

Common terms in the sports injury prevention literature	
Primary prevention	Reducing the risk for at-risk athletes [14]. Screening for risk factors [15]. Avoiding first time injury [16]. Preventing injury among athletes with no history of injury [17].
Secondary prevention	Reducing and minimizing injury severity [18]. Slowing or halting injury onset [19]. Preventing injury recurrence, reinjury or second injury [20]. Mitigating further associated complications [21].
Tertiary prevention	Reducing the effect of ongoing injury [22, 23]. Rehabilitating after injury [24]. Minimizing the consequences of injury [25, 26]. Reducing the risk of subsequent injury [27, 7].

**Fig. 1:**
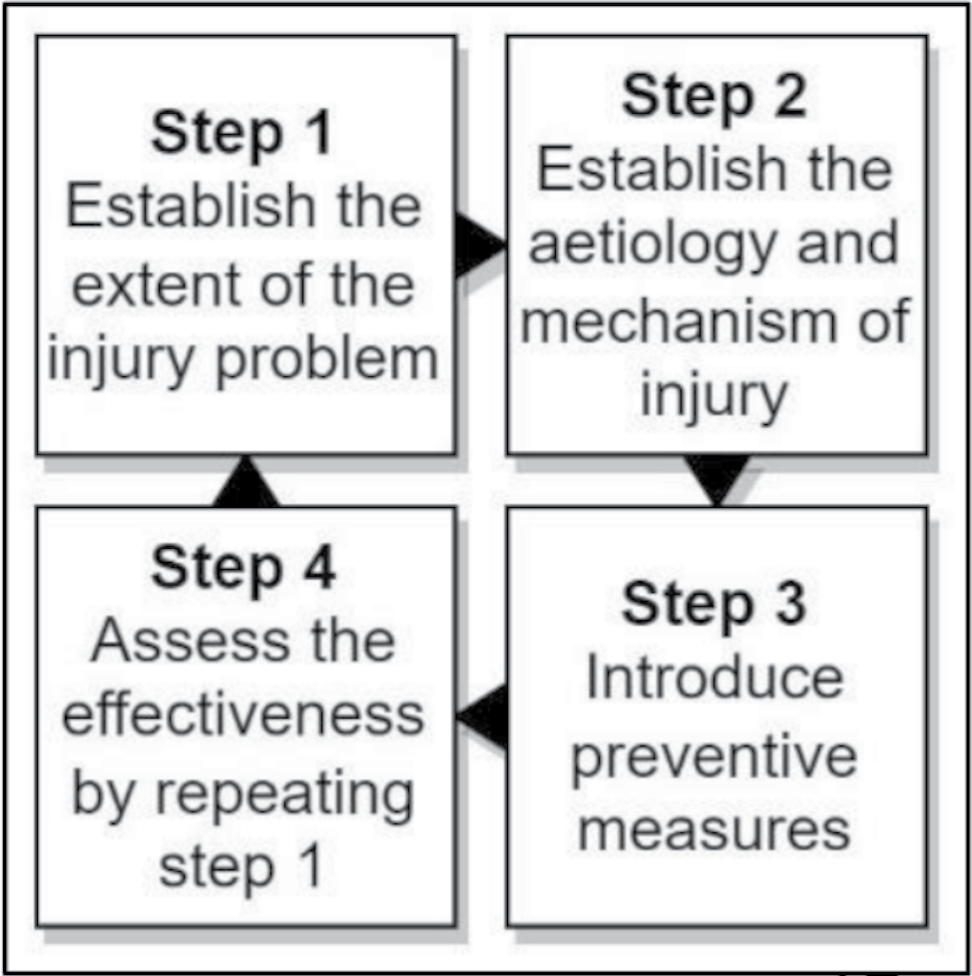
The ‘Sequence of prevention’ model reproduced with slight graphical changes from Bolling et al. [29].Originally distributed under the Creative Commons Attribution 4.0 International License (http://creativecommons.org/licenses/by/4.0/)

Frameworks developed for sport injury, such as the ‘the sequence of prevention model’ (Fig. 1), address the complexity of injury prevention in sport, and now offer a more context-specific approach for addressing injury prevention [28, 29]. However, as this model assumes equivalence between preventive measures (Step 3), scholars can potentially follow the same steps, yet reach different conclusions, depending on their interpretation of what constitutes primary, secondary or tertiary intervention.

The notion of validity as a criterion may be considered embedded throughout the research process. Consequently, conceptual clarity serves as the foundation for the cascade of methodological events that ultimately support or detract from research verisimilitude [30]. Furthermore, a lack of common terms of usage makes it difficult to compare and contrast investigations, and to develop a coherent body of evidence that moves the state-of-of-the-art forward.

Against this backdrop, our study aim was to systematically identify general terms of usage applied to prevention in the context of musculoskeletal sports injuries, in order to demonstrate whether these terms appear consistently across current intervention studies. Our aim was operationalized using the following research questions:

1. Are definitions used to define the concepts of primary, secondary and/or tertiary prevention, in the context of musculoskeletal sports injuries used consistently?
2. How can definitions of primary, secondary and tertiary prevention be focused in order to enhance the operationalizability of research questions in this context?

## Main Text

### Methods

We followed a rapid review method, a method following the systematic review procedure to synthesizing evidence in a timely and resource efficient manner for informing decisions in a health care setting [31]. The search strategy was carefully developed in collaboration with an expert librarian (also included in the author team) to secure rigor. We performed a title/abstract literature search in the following databases: Scopus, PubMed/Medline, Cochrane Library Reviews, Cochrane Library Trials, Web of Science, Sports Medicine and Education Index, SPORTDiscus and CINAHL. The search terms were *sport/athlete*, *injury*, *primary prevention*, *secondary prevention* and *tertiary prevention*, with the full search strings included in the supplementary material. To improve homogeneity of the preventive strategies, only articles on musculoskeletal injuries in sport or athletic context were included (for other inclusion/exclusion criteria, see Table 2). As sports injuries prevention is multidisciplinary (e.g., medical, surgical, public and allied health, etc.), we included several research fields. We used Covidence^®^ (i.e. an online software tool) to streamline the process of conducting the rapid review systematically, auto-removing duplicates before manual screening.

**Table 2:** Manuscript inclusion and exclusion criteria

Manuscript inclusion criteria:	Manuscript exclusion criteria:
Includes definition of primary, secondary and/or tertiary prevention. Athletes, all sports, all sexes, all ages. Musculoskeletal injuries. All manuscript types from the inception of the databases until November 2023.	Non-English. Non-athletes, excluding military personnel or manual laborers. Non-musculoskeletal injuries, excluding death, heart disease or brain injury.

A single researcher manually screened articles for full text review. After identification of relevant studies, three searches were performed in the text, searching for mentions of the *primary*, *secondary* or *tertiary* terms. We sought the researchers’ defined aim of each prevention class. If a prevention class was mentioned in an intervention study article’s title or keywords, but no specific definition was given within the article text, the study objectives were chosen as the definition of the prevention class aim. After extracting the researchers’ definition of each prevention class aim in quotes, a single researcher manually coded the quotes. The codes were adopted from Froom et al. (see Table 3) [1], and applied to each study. For example, in the quote: “Secondary prevention is also important and is needed to better treat these injuries so that long-term symptoms can be avoided and complications and risks of re-injuries can be reduced”, was coded as *Preventing Sequelae*, and *Preventing Recurrence* [32]. Finally, we summarized the results in a table adopted from Froom et al. [1]. The full dataset is available in the supplementary material.

**Table 3:** Description of included studies

Description of the included studies			
Publication type	Reviews	28	19%
	Commentaries	22	15%
	Clinical trials	21	15%
	Cohort studies	21	15%
	Cross-sectional studies	17	15%
	Other	35	24%
Publication date	2019–2023	75	52%
	2014–2018	49	34%
	Before 2013	20	14%
Sport setting	Football	13	9%
	Rugby	6	4%
	Other	25	17%
	Non-specified sport	103	72%
Injury	Anterior cruciate ligament rupture	29	20%
	Post-traumatic osteoarthritis	20	14%
	Ankle sprain	19	13%
	Hamstring strain	10	7%
	Other	23	16%
	Non-specified injury	43	30%
**Total studies**		**144**	

In an effort to aid future preventive intervention consistency and clarify the concepts of primary, secondary and tertiary prevention, we therefore subcategorized primary, secondary and tertiary prevention into six distinct aims of prevention.

## Results

**Fig. 2:**
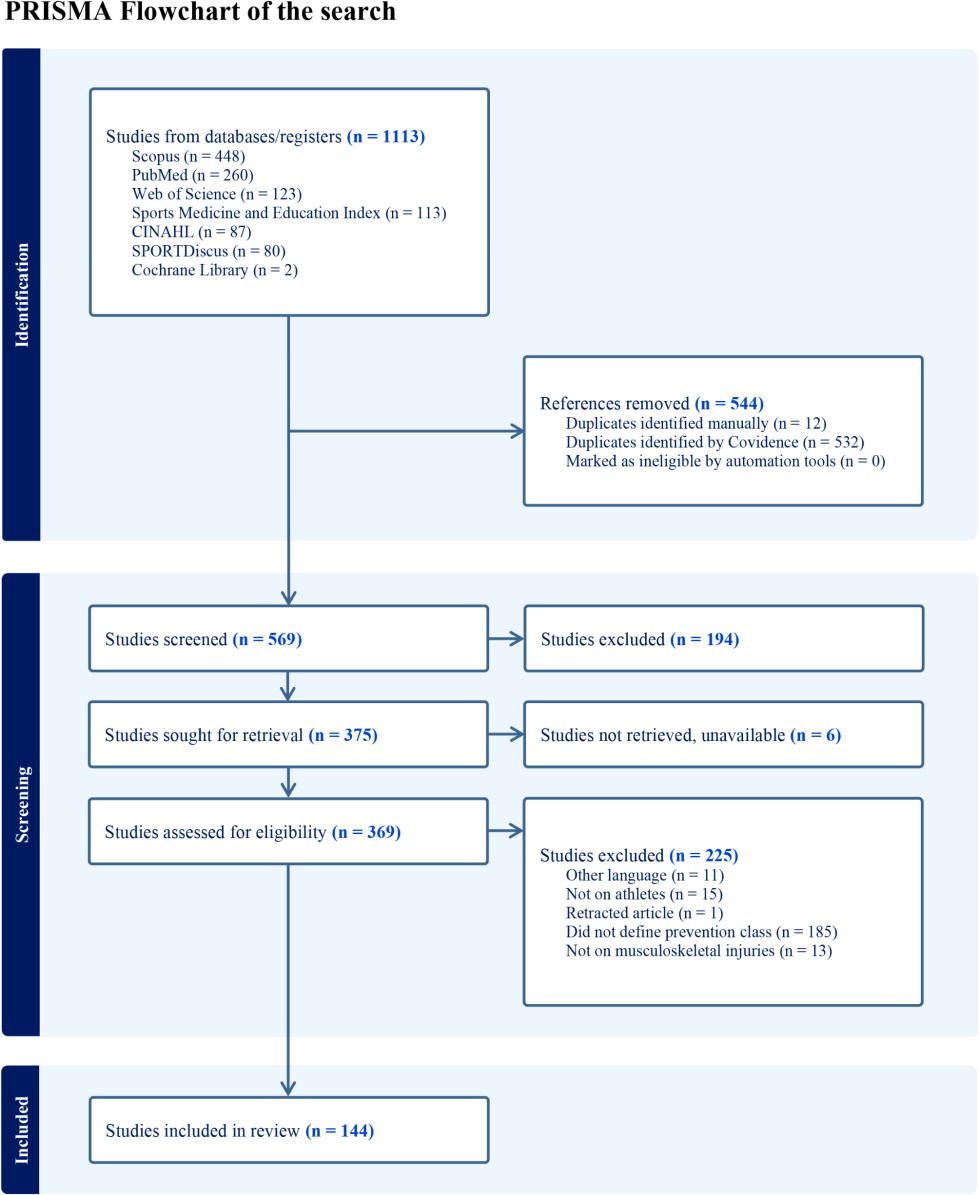
PRISMA flowchart of the search

The search identified 569 manuscripts after auto-removal of duplicates. After screening, 369 full text manuscripts were assessed for definition of prevention class definitions. A total of 144 studies reporting one or more definitions were included. The search is demonstrated in a PRISMA flowchart in Fig. 2.

The extracted definitions show that primary prevention appears focused on mitigating injury risk (*n* = 52) and preventing initial injuries (*n* = 42). Secondary prevention appears to address five distinct concepts: preventing recurrences (*n* = 42), preventing sequelae (*n* = 41), preventing index injury worsening (*n* = 27), mitigating injury risk (*n* = 15), and restoring function (*n* = 12). Tertiary prevention appears focused on preventing sequelae (*n* = 17) and restoring function (*n* = 9). The results are presented in Tables 4 and 3.

**Table 4:** Aim and class of prevention in the context of musculoskeletal sports injuries literature

		Publication classification^a^		
**Aim of prevention**	**Formal classification** ^**b**^	**Primary prevention**	**Secondary** **prevention**	**Tertiary** **prevention**
Avoiding the index injury	Primary	42 articles [33–35, 25, 36, 16, 12, 37–45, 17, 46, 47, 10, 48–53, 11, 54–57, 26, 58–63, 21, 64, 7, 23]	-	-
Mitigating injury risk	Primary	52 articles [65–70, 12, 71–74, 32, 75, 76, 15, 77, 42, 44, 78, 79, 19, 80, 81, 46, 82, 83, 11, 84, 85, 55, 86–89, 14, 90–92, 18, 93–105]	15 articles [66–69, 106, 39, 15, 107, 77, 53, 55, 108, 18, 109, 110]	4 articles [106, 15, 111, 27]
Preventing index injury worsening (early treatment)	Secondary	-	27 articles [112, 36, 16, 113, 114, 38, 39, 42, 44, 19, 115, 116, 46, 117–119, 54, 13, 86, 26, 59, 120, 21, 7, 23, 104, 105]	4 articles [77, 121, 18, 104]
Preventing injury sequelae	Secondary	1 article [74]	41 articles [122, 123, 25, 36, 113, 106, 124, 125, 32, 40, 126, 127, 107, 41, 42, 128, 45, 17, 116, 129, 130, 10, 131, 48, 50, 132, 51, 11, 133, 134, 56, 26, 108, 135, 14, 136, 61, 137, 138, 100, 139]	17 articles [106, 15, 42, 121, 44, 111, 140, 46, 53, 26, 141, 95, 21, 142, 7, 23, 105]
Preventing injury recurrence	Tertiary	-	42 articles [25, 143, 114, 144–148, 32, 39, 149, 41, 128, 150, 151, 80, 17, 130, 131, 48, 52, 22, 55, 56, 152, 58, 59, 135, 14, 9, 109, 60, 95, 153, 154, 62, 63, 100, 101, 20, 139, 155]	5 articles [36, 140, 53, 18, 27]
Restoring function (rehabilitation)	Tertiary	-	12 articles [41, 42, 151, 80, 131, 49, 22, 55, 95, 24, 139, 155]	9 articles [66, 69, 39, 156, 11, 59, 24, 21, 105]

^a^Publication classification is how the included articles in this rapid review define the aim of each prevention class

^b^Formal classification is how Brukner et al. [3] and most authors of epidemiological work [1] define the aim of each prevention class

## Discussion

Substantial inconsistencies were observed in grey areas between the prevention classes. This is most likely because the classes are theoretical attempts to categorize preventive interventions into discrete stages, while in clinical and athletic practice, prevention is likely to exist more as a continuum. For example, according to formal classification, an athlete may both be preventing a first-time ankle sprain (formally primary prevention), ankle instability after an ankle sprain (secondary prevention), and another ankle sprain (tertiary prevention) with the same intervention. It may also be argued that mitigating risk factors to prevent the initial injuries could be defined as primary prevention, and mitigating risk factors to prevent sequelae/recurrent injuries could be defined as secondary prevention.

While the classes intuitively seem to overlap with the normal-dysfunctional-pathological tissue continuum, such as in normal-reactive-degenerative tendinopathy [157, 158], our results indicate that the overlap is not clear cut. While primary prevention may be easily applied before the tissue becomes dysfunctional, both secondary and tertiary prevention are a range of interventions that may be applied from the onset of tissue dysfunction and onwards.

Despite our search strategy being designed to include studies on the adopted definition by clinicians working with athletes, we identified no such studies. As such, the results of the rapid review only demonstrate the definitions presented by the authors in the research literature, not the definitions adopted by clinicians in practice. Speculatively, the definitions presented by research authors may overlap with the definitions adopted by clinicians in practice, but observational research into this is required before such theorization may be substantiated.

Based on our results, it seems that researchers generally define the aim of primary prevention as: ‘Prevention of the initial injuries, and this includes mitigating injury risk factors’. Researchers define secondary prevention less consistently, both being internally inconsistent and overlapping with both primary and especially tertiary prevention.

Regarding *internal inconsistency*, the researchers largely disagree between preventing worsening, sequelae and/or recurrences. Some of this internal inconsistency may be because preventing worsening may be simply the strategy to prevent injury sequelae or recurrences. In studies on preventing traumatic musculoskeletal sports injuries (e.g., ankle sprain or anterior cruciate ligament injuries), secondary prevention is generally defined as prevention of recurrences, and in studies of insidious-onset musculoskeletal sports injuries (e.g., chronic ankle instability or post-traumatic knee osteoarthritis), secondary prevention is often defined as early detection/intervention. The results indicate that in the context of acute musculoskeletal sports injuries, it is impractical to prevent worsening by employing early treatment, which shifts the focus to preventing recurrences or injury sequelae instead.

Regarding *overlapping issues*, secondary prevention both overlaps with primary prevention (mentioned above), and some researchers define secondary prevention as restoring function, overlapping with tertiary prevention. This is also likely exacerbated by several studies referring to a second acute injury as a secondary injury, which likely has generated some confusion over what class of prevention prevents a second injury.

Based on our results, it seems that researchers generally define the aim of secondary prevention as: ‘Prevention of index injury worsening, injury sequelae and recurrences’, but overlapping somewhat with primary and tertiary prevention.

Tertiary prevention is comparatively less referenced in the literature than the other classifications, which Froom et al. [1] also noted in their similar study on medical conditions. When tertiary prevention is mentioned, it is often overlapping with secondary prevention. This overlapping is probably why tertiary prevention seems so rarely mentioned, as researchers often define such prevention as secondary prevention. It seems that researchers generally argue that the aim of tertiary prevention is ‘restoring function’, but overlapping somewhat with secondary prevention.

We highlighted clearly observable inconsistencies in the literature, which illustrate a problem in the generalization of research questions in the context of prevention of musculoskeletal sport injuries [159, 160]. Consequently, clinicians and researchers performing analyses on secondary prevention must be cognizant of variations such literature searches deliver. It is essential that a research field as a whole target the same phenomena. Such inconsistencies do not seem new in the broader sports literature, as exemplified by the similar inconsistencies of the definition of performance enhancement [161]. As injury prevention and performance enhancement overlap in new research, consistent definitions seem even more relevant [162].

**Fig. 3:**
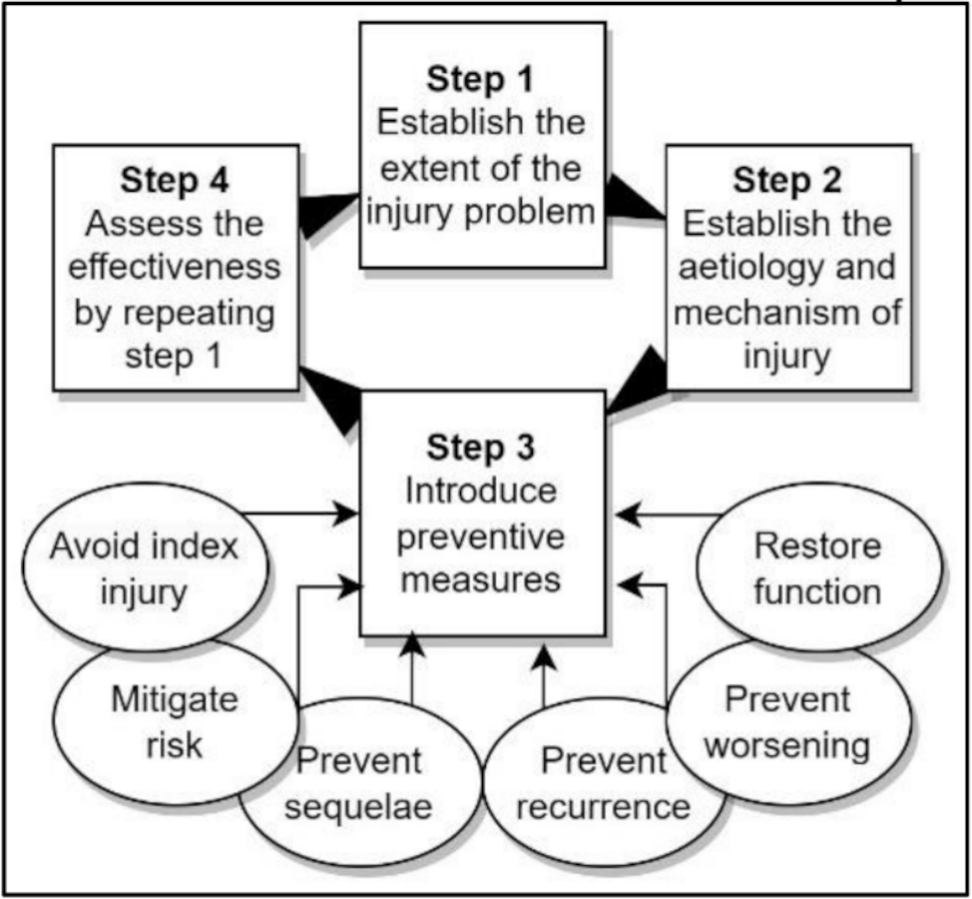
A modified ‘Sequence of Prevention’, expanded with details to Step 3 from Bolling et al. [29]. The inconsistently defined aims of the preventive measures demonstrate a central problem for researchers formulating research questions relating to primary, secondary or tertiary prevention. Originally distributed under the Creative Commons Attribution 4.0 International License (http://creativecommons.org/licenses/by/4.0/)

Based on our rapid review, we suggest to modify the ‘Sequence of Prevention’ model [29], in order to expand and detail Step 3, Introduce Preventive Measures (Fig. 3). This may guide researchers in formulating research questions pertaining primary, secondary or tertiary preventive interventions.

## Limitations

This rapid review has limitations that should be considered when interpreting the results. Firstly, we did not register a review protocol beforehand, and only one researcher performed the literature search, definition extraction, and definition coding. Employing a systematic review method, or having a second individual screening a sample of the studies to assess agreement statistics, would have strengthened the study. However, a 2008 study by Watt et al. concluded that rapid and full reviews did not differ extensively, suggesting that this is a valid and useful approach [163]. Nonetheless, to provide the study with transparency, we include the complete datasheet of the supplementary material.

Secondly, we did not apply controlled vocabulary (ex. MeSH-terms in PubMed) or include grey or non-English literature. These limitations reduced the number of included studies, but as the number of included studies was already high, it is unlikely that remedying these limitations would have changed the demonstrated inconsistency in the definition of prevention classes.

Our review did not include non-musculoskeletal diseases to homogenize prevention intervention strategies. It is possible that prevention of these conditions is more or less consistently defined, but the work of Froom et al. [1] suggests that the concepts are just as inconsistent in the general field.

To ensure that focus was on sport injuries in general and identify as many definitions of musculoskeletal sport injuries prevention aims as possible, we did not limit our search to specific research fields (e.g., medical, surgical, physiotherapeutic, etc.), country, age or type of sport. It is possible that analyses within these subgroups may have demonstrated concept consistency variance between the fields. For such research, our data is available in the supplementary material appendix.

## Conclusion

Substantial heterogeneity in the choice of definition and reporting of primary, secondary and tertiary prevention was evident in the sports injuries research literature. This undermines preventive intervention consistency and consequently represents a threat to the internal validity of the interventions. A greater degree of uniform and nuanced reporting is required to improve research verisimilitude of primary, secondary and tertiary preventive measures. To address this issue, we recommend the implementation of fully articulated working definitions of the aim of preventive interventions.

Our findings support the definition of primary prevention aim to be preventing initial injuries and mitigating risk factors. Secondary prevention aim is defined more inconsistently, generally as preventing injury worsening, sequelae and recurrences, but also overlapping heavily with primary and tertiary prevention. Finally, tertiary prevention is less referenced in the literature, its aim primarily defined as to restore function, but also heavily overlapping with secondary prevention. These suggested working definitions of the aim of prevention should guide future researchers formulating research questions.

## Electronic Supplementary Material

Below is the link to the electronic supplementary material.

Supplementary Material 1

## Data Availability

Being a literature review, the data are available in the cited manuscripts– however, the dataset supporting the conclusions of this article is included within the article and its supplementary material.
